# Sofosbuvir/velpatasvir is an effective treatment for patients with hepatitis C and advanced fibrosis or cirrhosis in a real-world setting in Taiwan

**DOI:** 10.1186/s12876-021-01837-y

**Published:** 2021-06-12

**Authors:** Yu-Ting Huang, Yung-Yu Hsieh, Wei-Ming Chen, Shui-Yi Tung, Kuo-Liang Wei, Chen-Heng Shen, Kao-Chi Chang, Chung-Kuang Lu, Chih-Wei Yen, Sheng-Nan Lu, Chao-Hung Hung, Te-Sheng Chang

**Affiliations:** 1 Division of Gastroenterology and Hepatology, Department of Internal Medicine, Chang Gung Memorial Hospital, No. 6, Section West, Chiapu Road, Puzi, Chiayi 613 Taiwan; 2 College of Medicine, Chang Gung University, Taoyuan, Taiwan

**Keywords:** Sofosbuvir/velpatasvir, Hepatitis C, Liver cirrhosis, Sustained virologic response

## Abstract

**Introduction:**

Real-world data regarding the impact of hepatic fibrosis on the effectiveness of sofosbuvir/velpatasvir (SOF/VEL) treatment is limited in the Asian population.

**Methods:**

We analyzed data for all 823 patients with hepatitis C virus treated with SOF/VEL from June 2019 to September 2020 at Chang Gung Memorial Hospital in Chiayi, Taiwan. The degree of fibrosis was determined using the fibrosis-4 (FIB-4) index, with advanced fibrosis or cirrhosis defined as a FIB-4 score of > 3.25. The primary treatment outcome was the rate of sustained virologic response 12 weeks after treatment cessation (SVR). Adverse events (AEs) were also evaluated.

**Results:**

SVR rates did not significantly differ (*p* > 0.05) between patients with FIB-4 scores of ≤ 3.25 and those with scores of > 3.25. In the per protocol analysis, 99.2% (593/598) of the FIB-4 ≤ 3.25 group and 100% (172/172) of the FIB-4 > 3.25 group achieved SVR; in the evaluable population analysis, 93.4% (593/635) of the FIB-4 ≤ 3.25 group and 91.5% (172/188) of the FIB-4 > 3.25 group achieved SVR. Five patients with FIB-4 scores of ≤ 3.25 did not attain SVR: two relapsed and three had no response. The most common AEs were comparable (*p* > 0.05) for the FIB-4 ≤ 3.25 group and the FIB-4 > 3.25 group and included abdominal discomfort (4.4% vs. 5.9%), fatigue (4.1% vs. 5.9%), and skin itching (3.6% vs. 3.2%). Laboratory abnormalities were more common in the FIB-4 > 3.25 group (*p* < 0.001). Six patients with FIB-4 scores of > 3.25 had total bilirubin elevation > 3 × the upper normal limit (UNL). Alanine transaminase elevation > 5 × the UNL was observed in two patients with FIB-4 scores of ≤ 3.25 and one patient with a FIB-4 score of > 3.25. No AEs resulted in treatment discontinuation.

**Conclusions:**

SOF/VEL treatment is well tolerated and achieves high SVR rates for patients of Taiwanese ethnicity with HCV, regardless of cirrhosis status.

**Supplementary Information:**

The online version contains supplementary material available at 10.1186/s12876-021-01837-y.

## Introduction

Hepatitis C virus (HCV) infection has long been a major global public health problem; it is not only a leading cause of end-stage liver disease but also results in increased mortality rates for many extrahepatic diseases [1, 2]. In 2015, estimates indicated that approximately 110 million people had a history of HCV infection and 71.1 million people (1% of the global population) were living with active viremic infection [3, 4]. Due to a lack of effective vaccines, patients with HCV must attain a sustained virologic response (SVR) for improved long-term outcomes. SVR is defined as an undetectable serum HCV RNA level 12 weeks posttherapy for patients treated with interferon-free direct-acting antiviral (DAA) therapy or 24 weeks posttherapy for patients treated with interferon-based therapy [5].

The advent of new highly effective and well-tolerated DAAs has had a considerable effect on HCV treatment [6]. The advancement in HCV therapeutics has led to SVR rates reaching 95–99% across all HCV genotypes, bringing the prospect of eliminating HCV as a public health threat into sight [7]. Despite the high effectiveness of such treatment, a small proportion of patients with HCV (< 5%) still experience virological failure following DAA regimens [8]. Liver cirrhosis is a key factor among the common viral and host factors associated with DAA failure, especially for patients receiving sofosbuvir (SOF)-based therapies [8–10].

SOF is a liver-targeted pyrimidine nucleotide analog with pan-genotypic potency, which works as a chain terminator that inhibits HCV nonstructural protein 5B (NS5B) RNA-dependent RNA polymerase. Velpatasvir (VEL) is a new-generation NS5A inhibitor with pan-genotypic antiviral activity. The fixed-dose combination of SOF/VEL (400 mg/100 mg, Epclusa, Gilead Sciences) is the first pan-genotypic DAA to exhibit high effectiveness and safety profiles in serial clinical trials; it was approved to treat HCV genotypes 1–6 in patients with or without compensated cirrhosis, with the addition of ribavirin recommended in the presence of decompensated cirrhosis [11].

A large-scale real-world study of 5552 patients from 12 clinical practice cohorts across Europe and North America demonstrated SOF/VEL was highly effective; only 1% of patients did not achieve SVR, and liver cirrhosis was the only factor associated with an increased risk of not achieving SVR [12]. In Asia, a phase 3 clinical trial of 375 patients with HCV also demonstrated a high SVR rate of 97%, but lower efficacy was observed in those with cirrhosis [13]. By contrast, another real-world population cohort study consisting of 2,821 patients in Canada discerned no impact of cirrhosis on SVR [14]. In light of these conflicting results and the limited real-world data in Asia, we assessed the impact of advanced fibrosis and cirrhosis on SOF/VEL effectiveness for patients with HCV in Taiwan.

## Patients and methods

### Patients

With the availability of all-oral DAAs, the Taiwan National Health Insurance (NHI) Administration initiated a nationwide government-funded program for treating chronic HCV infection with DAAs in 2017 as a part of an effort to eliminate HCV by 2025 [15]. The program enrolled patients with concurrent chronic HCV infection and advanced fibrotic or cirrhotic liver disease from 2017 to 2018. In 2019, the program was extended to all patients with HCV and active viremic infection, regardless of the duration or severity of liver disease. The only exclusion criterion was having an advanced or terminal stage disease with a life expectancy of < 6 months. SOF/VEL was approved by the Taiwan Food and Drug Administration (TFDA) on December 17, 2018, and began being reimbursed by Taiwan NHI on June 1, 2019, for patients with chronic HCV genotype 1–6 infection. This study enrolled patients with HCV of all various genotypes who were ≥ 20 years old and received treatment with SOF/VEL from June 2019 to September 2020 at Chang Gung Memorial Hospital in Chiayi, Taiwan.

### Study design

Baseline patient demographic data and on-treatment information, including laboratory changes and adverse events (AEs), were obtained from the electronic medical records, as described previously [16]. Treatment for patients with HCV of various genotypes with a fixed-dose combination of SOF/VEL was determined at the discretion of the treating physician on the basis of the labels approved by the TFDA, in compliance with the standard of care recommended by international guidelines for HCV infection [17]. Briefly, SOF/VEL was prescribed for a duration of 12 weeks to all patients, and ribavirin was added for patients with a history of liver decompensation as indicated by Child–Pugh-Turcotte (CPT) class B or C. The degree of hepatic fibrosis was assessed using the fibrosis-4 (FIB-4) index, which is calculated using the following formula: age (years) × AST [U/L]/(platelets [10^9^/L] × (ALT [U/L])^1/2^). A FIB-4 score of > 3.25 denotes advanced fibrosis or cirrhosis (F3–F4) [18]. All patients gave written informed consent prior to the initiation of DAA therapy. This study was approved by the Institutional Review Board of Chang Gung Medical Foundation and was conducted in accordance with the principles of the Declaration of Helsinki and the International Conference on Harmonization for Good Clinical Practice.

### Outcome evaluation

The primary outcome was the rate of SVR, which was defined as the proportion of patients with serum HCV RNA levels under the lower limit of detection (LLOD) 12 weeks after treatment cessation, as determined by per protocol (PP) analysis (participants who received ≥ 1 dose of DAA with HCV RNA data at posttreatment week 12) or evaluable population (EP) analysis (participants who received ≥ 1 dose of DAA with at least one available postbaseline response assessment). Secondary outcomes included AE incidence and the rate of undetectable serum HCV RNA levels (< LLOD) at the end of treatment (EOT).

### Statistical analysis

Statistical analyses were performed using SPSS Statistics (version 22.0; IBM, Chicago, IL, USA). Continuous variables were expressed as means ± standard deviations or median and range. Descriptive characteristics were expressed as numbers (percentages) for the categorical variables. Differences between groups were analyzed using the chi-square test for categorical variables and Student’s *t* test or Mann–Whitney *U* test for continuous variables as appropriate. The Kruskal–Wallis test was used to compare the intergroup differences among CPT classes. A two-tailed *p* value of < 0.05 was considered statistically significant.

## Results

### Patient baseline characteristics

**Fig. 1:**
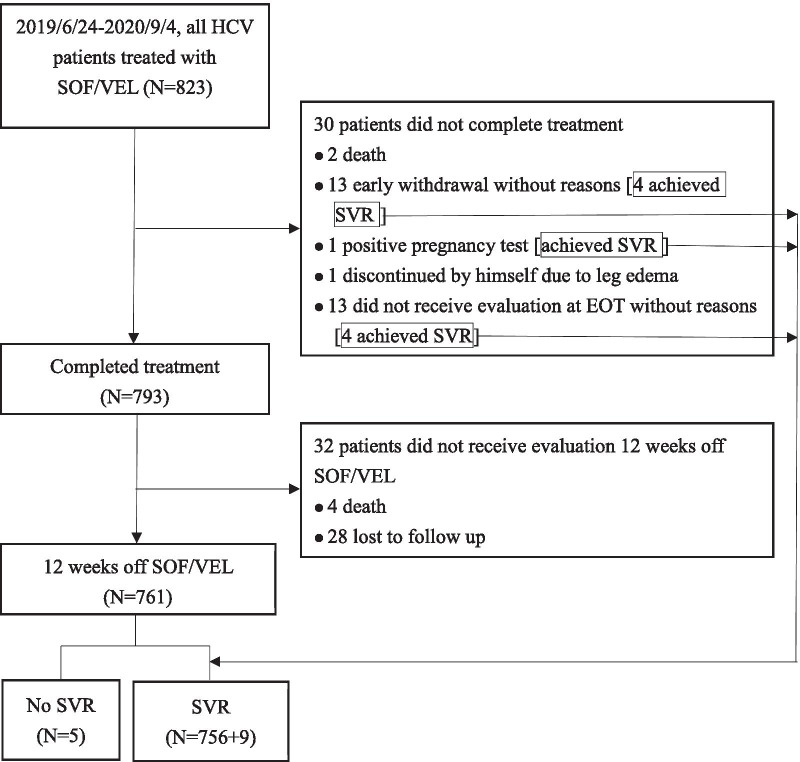
Study flow diagram

We enrolled all 823 patients with HCV who received SOF/VEL treatment at Chiayi Chang Gung Memorial Hospital between June 2019 and September 2020. As shown in Fig. 1, 30 patients did not complete treatment: 2 died, 1 had a positive pregnancy test, 1 was diagnosed with leg edema, 13 withdrew early for unknown reasons, and 13 failed to receive an evaluation at EOT. Among these 30 patients, 9 attained SVR. Of the 793 patients who completed SOF/VEL treatment, 32 failed to undergo an evaluation 12 weeks after cessation of SOF/VEL treatment due to four deaths and 28 losses to follow-up.

Of the 823 patients, 188 patients had FIB-4 scores of > 3.25, indicating advanced fibrosis or cirrhosis. Table 1 displays the baseline characteristics of these 823 patients. Compared to those with a FIB-4 score of > 3.25, participants with a FIB-4 score of ≤ 3.25 were younger (61.87 ± 14.33 vs. 70.53 ± 12.75 years, *p* < 0.001); had lower incidences of ribavirin use (*p* < 0.001) and prior hepatocellular carcinoma (HCC) (*p* < 0.001); had higher levels of HCV RNA (2,261,880 [17–39,902,035] vs. 1,047,236 [51–31,094,685] IU/mL, *p* < 0.001), hemoglobin (13.67 ± 1.8 vs. 12.82 ± 2.08 g/dL, *p* < 0.001), albumin (4.29 ± 0.35 vs. 3.85 ± 0.51, *p* < 0.001), and platelet count (218.80 ± 66.93 vs. 129.14 ± 51.23 × 10^9^ cells/L, *p* < 0.001); and had lower levels of AST (38.83 ± 24.84 vs. 95.62 ± 68.27 U/L, *p* < 0.001), ALT (51.69 ± 56.24 vs. 97.24 ± 88.12 U/L, *p* < 0.001), total bilirubin (0.84 ± 0.64 vs. 1.27 ± 1.01, *p* < 0.001), and alpha-fetoprotein (3 [0.7–114.1] vs. 5.7 [1.2–52,225.3] ng/mL, *p* < 0.001). There is no surprise that significant differences were observed in the baseline CPT class for the FIB-4 scores of ≤ 3.25 vs. > 3.25 groups (CPT class A/B/C: 632/3/0 vs. 164/23/1, *p* < 0.001). No differences were evident in gender; renal function; or incidence treatment history, HBV coinfection, or diabetes mellitus between the two groups (*p* > 0.05). The most common HCV genotypes including genotype 2 (N = 404, 49.09%), genotype 1b (N = 326, 39.61%), mixed genotype (N = 41, 4.98%), and genotype 1a (N = 27, 3.28%).

**Table 1:** Baseline characteristics of patients

	FIB-4 ≤ 3.25	FIB-4 > 3.25	*p* value
Patient number	635	188	
Age, year			
Mean ± standard deviation (SD)	61.87 ± 14.33	70.53 ± 12.75	< 0.001
Sex			
Male/female	309/326	99/89	0.38
Treatment experience			
Naïve/experienced	605/30	181/7	0.7
HBV coinfection			
Absent/present	565/70	173/15	0.29
Prior HCC history			
No/yes	627/8	154/34	< 0.001
Ribavirin usage			
No/yes	631/4	162/26	< 0.001
Child–Pugh–Turcotte class			
A/B/C	632/3/0	164/23/1	< 0.001
Diabetes mellitus			
No/Yes	509/126	143/45	0.27
HCV RNA, IU/mL			
< 800,000/ ≥ 800,000	195/440	80/108	< 0.05
HCV RNA, IU/mL, median (range)	2,261,880 (17–39,902,035)	1,047,236 (51–31,094,685)	< 0.001
Hemoglobin, g/dL, mean ± SD	13.67 ± 1.8	12.82 ± 2.08	< 0.001
White blood cell count, 10^9^cells/L, mean ± SD	6.77 ± 2.06	5.8 ± 6.69	0.05
Platelet count, 10^9^cells/L, mean ± SD	218.80 ± 66.93	129.14 ± 51.23	< 0.001
Albumin, g/dL, mean ± SD	4.29 ± 0.35	3.85 ± 0.51	< 0.001
Total bilirubin, mg/dL, mean ± SD	0.84 ± 0.64	1.27 ± 1.01	< 0.001
AST, U/L, mean ± SD	38.83 ± 24.84	95.62 ± 68.27	< 0.001
ALT, U/L, mean ± SD	51.69 ± 56.24	97.24 ± 88.12	< 0.001
Creatinine, mg/dL, mean ± SD	1.00 ± 1.04	0.96 ± 0.54	0.53
eGFR, mL/min/1.73m^2^, mean ± SD	85.77 ± 26.23	82.55 ± 28.28	0.15
Alpha-fetoprotein, ng/mL, median (range)	3 (0.7–114.1)	5.7 (1.2–52,225.3)	< 0.001

HCV genotype	Patient number	Patient number	Total number
1a	19	8	27 (3.28%)
1b	267	59	326 (39.61%)
2	301	103	404 (49.09%)
3	2	2	4 (0.49%)
6	14	4	18 (2.19%)
Mixed	30	11	41 (4.98%)
No data	2	1	3 (0.36%)

### Effectiveness outcome

**Fig. 2:**
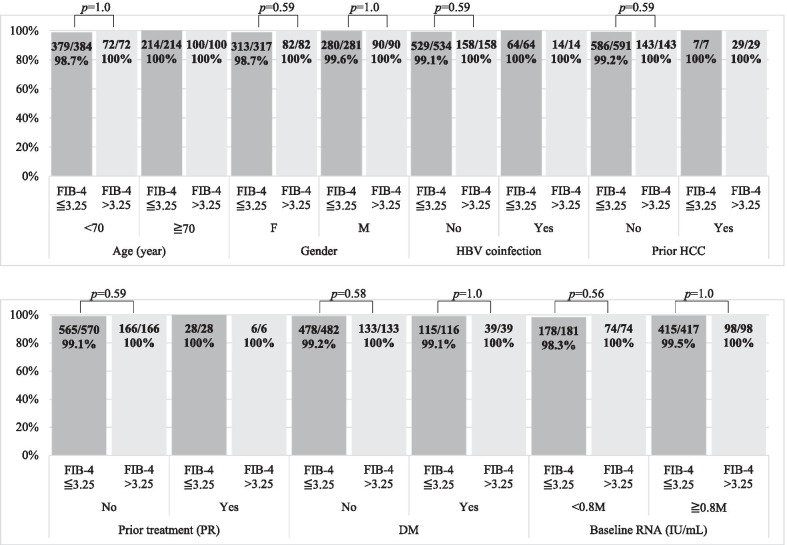
Subgroup analysis of SVR (per protocol set)

As shown in Table 2, the response rates at EOT were 99.5% (609/612) for the FIB-4 ≤ 3.25 group and 100% (183/183) for the FIB-4 > 3.25 group (*p* = 0.79). No difference was identified in the rate of SVR between the FIB-4 ≤ 3.25 and FIB-4 > 3.25 groups. According to the PP analysis, the SVR rates were 99.2% (593/598) for the FIB-4 ≤ 3.25 group and 100% (172/172) the FIB-4 > 3.25 group (*p* = 0.51); according to the EP analysis, SVR rates were 93.4% (593/635) for the FIB-4 ≤ 3.25 group and 91.5% (172/188) for the FIB-4 > 3.25 group (*p* = 0.47). All five patients who did not attain SVR had FIB-4 scores of < 3.25, including three nonresponses and two relapses. The detailed baseline characteristics of the five patients who did not attain SVR are displayed in Table 3. Subgroup analyses conducted for age, gender, HBV coinfection, diabetes mellitus, prior HCC, prior treatment experience, and baseline HCV RNA levels revealed no differences in effectiveness between the FIB-4 ≤ 3.25 and FIB-4 > 3.25 groups (*p* > 0.05; Fig. 2).

**Table 2:** Virological responses

HCV RNA < LLOD	FIB-4 ≤ 3.25 (N = 635), N/N (%)	FIB-4 > 3.25 (N = 188), N/N (%)	*p* value
End of treatment			
ETR (PP)	609/612 (99.5)	183/183 (100)	0.79
Lost to follow-up	23	5	
After treatment			
SVR (EP)	593/635 (93.4)	172/188 (91.5)	0.47
SVR (PP)	593/598 (99.2)	172/172 (100)	0.51
Reason for non-SVR_,_ N			
Relapse	2	0	
Nonresponse	3	0	
Lost to follow-up	37	16	

EP, evaluable population; ETR, end-of-treatment response; PP, per protocol; SVR, sustained virological response

**Table 3:** Baseline characteristics of patients without SVR

	Case 1	Case 2	Case 3	Case 4	Case 5
Type of non-SVR	Relapse	Relapse	Nonresponse	Nonresponse	Nonresponse
Ribavirin usage	No	No	No	No	No
Prior treatment	No	No	No	No	No
Gender	Male	Female	Female	Female	Female
Age, years	59	57	36	62	40
BMI, kg/m^2^	28.3	19.5	20.9	21.6	33.4
DM	Yes	No	No	No	No
HBV coinfection	No	No	No	No	No
Prior HCC	No	No	No	No	No
Cirrhosis	No	No	No	No	No
Fib-4 score	0.86	1.45	0.67	1.48	1.09
Genotype	2	2	1b	1b	2
HCV RNA, IU/ml	17	8,339,147	22,047	1,293,629	335,076
AST, U/L	21	28	21	20	70
ALT, U/L	29	22	22	14	124
Total bilirubin, mg/dl	0.4	0.5	0.6	0.9	0.4
Direct bilirubin, mg/dl	0.2	0.2	0.1	0.1	0.1
Creatinine, mg/dl	1.18	0.83	0.57	0.91	0.53
eGFR, ml/min/1.73 m^2^	67.15	75.31	127.56	66.58	135.8
Albumin, g/dl	4.4	4.8	4	4.3	4.5
WBC, 10^9^ cells/L	8.8	6.5	5.2	4.9	5.3
Hemoglobin, g/dl	13.5	13.8	12.5	12.9	13.1
Platelet, 10^9^ cells/L	269	234	241	224	230
INR	1.02	0.98	1.02	1.02	1.06

### Safety outcomes

A total of 793 patients completed SOF/VEL treatment (Fig. 1). Both patients who did not complete SOF/VEL treatment due to death died during week 12; one death resulted from occult infection with hepatic encephalopathy, and the other resulted from spontaneous bacterial peritonitis with *Staphylococcus aureus* and *Salmonella* bacteremia. Of the four patients who died after completing SOF/VEL treatment but failed to undergo SVR evaluation, one died during posttreatment week 5 due to pneumonia and septic shock, one died during posttreatment week 6 due to acute respiratory failure and cardiac arrest, one died during posttreatment week 7 due to suspected acute myocardial infarction, and one died during posttreatment week 9 due to pneumonia or lung malignancy. None of the six deaths were considered attributable to SOF/VEL treatment.

As listed in Table 4, the most common AEs (those affecting > 3% of all patients), were similar for the FIB-4 ≤ 3.25 and FIB-4 > 3.25 groups and included abdominal discomfort (4.4% vs. 5.9%, *p* = 0.53), fatigue (4.1% vs. 5.9%, *p* = 0.41), and skin itching (3.6% vs. 3.2%, *p* = 0.96). SOF/VEL-associated laboratory abnormalities were more common in the FIB-4 > 3.25 group (*p* < 0.001). Clinically significant laboratory abnormalities were rare: in the FIB-4 > 3.25 group, only six patients had a > 3 × elevation of total serum bilirubin level, two patients had a ˃5 × elevation of serum AST level, and one patient had a ˃5 × elevation in serum ALT level; in the FIB-4 ≤ 3.25 group, two patients had a ˃5 × elevation in serum ALT level. None of these AEs resulted in the discontinuation of SOF/VEL treatment.

**Table 4:** Safety summary

Event, N (%)	FIB-4 ≤ 3.25 (N = 635)	FIB-4 > 3.25 (N = 188)	*p* value
Adverse events, > 3%	77 (12.1)	28 (15)	*0.38*
Abdominal discomfort	28 (4.4)	11 (5.9)	*0.53*
Fatigue	26 (4.1)	11 (5.9)	*0.41*
Skin itching	23 (3.6)	6 (3.2)	*0.96*
Laboratory adverse event	14 (2.2)	32 (17)	< 0.001
Total bilirubin elevation	6 (0.9)	21 (11.2)	< 0.001
1.5–3 × ULN	6 (0.9)	15 (8)	< 0.001
> 3 × ULN	0	6 (3.2)	< 0.001
AST elevation	3 (0.5)	8 (4.3)	< 0.001
3–5 × ULN	3 (0.5)	6 (3.2)	< 0.001
> 5 × ULN	0	2 (1.1)	*0.05*
ALT elevation	5 (0.8)	3 (1.6)	*0.39*
3–5 X ULN	3 (0.5)	2 (1.1)	0.32
> 5 × ULN	2 (0.3)	1 (0.5)	*0.54*

*p* value in italics indicates statistical nonsignificance between the two groups

## Discussion

Treatment for HCV has evolved rapidly since 2014, from long-course, interferon-based regimens with suboptimal efficacy and substantial AEs to short-course, well-tolerated, and highly effective all-oral DAA therapy [19]. In 2017, this advance motivated the World Health Organization to update its guidelines for the screening, care, and treatment of persons with HCV infection and to establish the ambitious goal of HCV elimination by 2030 [7]. The emergence of pan-genotypic DAAs can further accelerate the realization of the HCV elimination goal by simplifying the treatment algorithms, as resource-intensive genotyping and frequent laboratory monitoring can be eliminated [20].

The fixed-dose combination of SOF/VEL is the first approved pan-genotypic DAA with well-established effectiveness and safety profiles from both clinical trials and real-world settings [12–14, 21]. Compared with protease inhibitor (PI)-containing DAA regimens, the PI-free SOF/VEL regimen has several advantages: it involves a lower pill burden, has fewer potential drug interactions, and can be administered to patients with decompensated cirrhosis and renal failure [22, 23]. Furthermore, treatment with SOF/VEL has been reported to be cost effective and to improve health-related quality of life [24, 25]. However, current data regarding the effects of SOF/VEL in the Asian population are limited, as the ASTRAL 1–5 trials investigating the effectiveness of SOF/VEL on various HCV genotypes or special populations enroll primarily white patients, and most of the studies published to date were conducted in Western countries [12, 14, 21, 26].

Liver cirrhosis is a major factor associated with decreased SVR rates, especially for sofosbuvir-based regimens [8–10]. However, real-world data regarding the impact of hepatic fibrosis on the effectiveness of SOF/VEL are limited for the Asian population. The present study, composed entirely of Taiwanese patients, revealed similarly high effectiveness of SOF/VEL for patients with HCV and advanced fibrosis or cirrhosis (FIB-4 score ≤ 3.25 or F3–F4) compared with their counterparts with mild or no liver fibrosis (FIB-4 score ≤ 3.25 or F0–F2). Our results were consistent with another report by Asselah et al., in which SOF/VEL exhibited high effectiveness and safety profiles for treating various genotypes of HCV in patients, of whom only 8% were of Asian descent, with advanced fibrosis or compensated cirrhosis [26].

Determining the degree of liver fibrosis is essential but challenging for the assessment and management of patients with chronic liver disease. Each tool used to evaluate the degree of liver fibrosis has shortcomings, and it is difficult to make comparisons between the various modalities [27]. In contrast to Asselah et al.’s study, which employed the expensive and difficult to access FibroTest, FibroScan, or liver biopsy to assess liver fibrosis, our study used the FIB-4 index to define the degree of advanced fibrosis. The FIB-4 score is simple and inexpensive because it is calculated by incorporating four readily available parameters: age, AST, ALT, and platelet count [18]. This index exhibited satisfactory diagnostic performance for assessing liver fibrosis in Asian patients with chronic viral hepatitis B and C [28]. The FIB-4 score was also shown to be superior to the CPT class and MELD score in predicting the long-term outcomes of patients with HCV [29].

Because the addition of ribavirin to SOF/VEL combination in patients with cirrhosis has been debatable and is of particular interest, the details regarding the ribavirin dosage and treatment adherence of the 30 patients (baseline CPT class A/B/C: 3/26/1) who received ribavirin were demonstrated in Additional file 1: supplementary Table 1. Among the 30 patients treated with SOF/VEL plus ribavirin, 4 patients prematurely discontinued treatment (2 without reasons, 1 died of spontaneous bacterial peritonitis with bacteremia and 1 lost to follow-up) and 3 patients attained end-of-treatment response (ETR) but did not complete the post-treatment follow-up (2 died of causes unrelated to liver disease and 1 without reasons). All these 7 patients belonged to CPT class B at baseline. In our real-world cohort, ribavirin was given in a low dose ranging from 200 to 1000 mg daily. Dose adjustment was required in 11 patients despite the relatively low ribavirin dosage, mostly due to anemia.

As demonstrated in Fig. 1, a total of 62 patients did not complete the treatment protocol with 30 prematurely discontinued treatment and 32 did not complete the post-treatment follow-up. Of these 62 patients, 7 received ribavirin comprising 23.3% of all the 30 patients receiving ribavirin. The percentage of 23.3% non-adherence for the 30 patients with ribavirin use was much higher than that of 6.9% for patients without ribavirin use which consisted 55 non-adherence out of the 793 ribavirin-free patients. Even though, it is difficult to conclude that ribavirin use contributed to the decreased treatment adherence.

Our study has several limitations. First, selection bias may have occurred as our patients were enrolled from a single referral center. Second, because this was a retrospective study, mild to moderate AEs might have been underreported. Third, as mentioned earlier, each modality for evaluating liver fibrosis has certain shortcomings.

In conclusion, our study demonstrated that SOF/VEL regimens are safe and achieve high SVR rates for Asian patients with HCV infection, regardless of cirrhosis status.

## Supplementary Information

**Additional file 1**. Supplementary Table 1. Ribavirin dosage and treatment adherence of the 30 patients treated with SOF/VEL plus ribavirin.

## Data Availability

The datasets used and/or analysed during the current study available from the corresponding author on reasonable request.

## Abbreviations

AE: Adverse event
ALT: Alanine aminotransferase
AST: Aspartate aminotransferase
CPT: Child–Pugh–Turcotte
DAA: Direct-acting antiviral agent
eGFR: Estimated glomerular filtration rate
EOT: End of treatment
ETR: End-of-treatment response
EP: Evaluable population
FIB-4: Fibrosis index based on four factors
HCC: Hepatocellular carcinoma
LLOD: Lower limit of detection
NHI: National Health Insurance
NS5B: Nonstructural protein 5B
PI: Protease inhibitor
PP: Per protocol
SVR: Sustained virologic response 12 weeks after treatment cessation
TFDA: Taiwan Food and Drug Administration
