# Supplementary material for: Sofosbuvir/velpatasvir is an effective treatment for patients with hepatitis C and advanced fibrosis or cirrhosis in a real-world setting in Taiwan
Source: BMC Gastroenterol. 2021 Jun 12;21:259. doi: 10.1186/s12876-021-01837-y (PMC8199354; doi:10.1186/s12876-021-01837-y)
Supplement: Supplementary file 1 — Additional file 1. Supplementary Table 1. Ribavirin dosage and treatment adherence of the 30 patients treated with SOF/VEL plus ribavirin. [file 12876_2021_1837_MOESM1_ESM.docx]

**Supplementary Table 1. Ribavirin dosage and treatment adherence of the 30 patients treated with SOF/VEL plus ribavirin**

| CTP class | Ribavirin dose adjustment | Ribavirin dosage | Total (N) | SVR (n) | Reasons of treatment withdrawal (number of withdrawal = 7) |
| --- | --- | --- | --- | --- | --- |
| A | No | 400mg/day | 1 | 1 |  |
|  | Yes | W1: 600mg/day, W2-W6: 1000mg/day, W7-W12: 800mg/day for anemia | 1 | 1 |  |
|  |  | W1: 200mg/day, W2-W12: no ribavirin | 1 | 1 |  |
| B | No | 400mg/day | 3 | 1 | One attained ETR, but died at post treatment week 9 due to pneumonia or lung malignancy, another did not receive ETR and SVR evaluation without reasons |
|  |  | 600mg/day | 2 | 2 |  |
|  |  | 800mg/day | 8 | 7 | One withdrew due to admission at another hospital |
|  |  | 1000mg/day | 4 | 4 |  |
|  | Yes | W1-W4: 1000mg/day, W5-W6: 800mg/day for anemia | 1 | 0 | Withdrew after week 6 without reasons |
|  |  | W1-W8: 1000mg/day, W9-W10: 800mg/day, W11-W12: 600mg/day for anemia | 1 | 0 | Attained ETR, but did not receive SVR evaluation without reasons |
|  |  | W1-W4: 800mg/day, W5-W6: 400mg/day for anemia | 1 | 0 | Withdrew without reasons and died at week 12 due to spontaneous bacterial peritonitis. |
|  |  | W1-W4: 800mg/day, W5-W12:1000mg/day | 1 | 1 |  |
|  |  | W1-W2: 800mg/day, W3-W12: 400mg/day for anemia | 1 | 1 |  |
|  |  | W1-W6: 600mg/day, W7-W12: 200mg/day for anemia | 1 | 1 |  |
|  |  | W1-W5: 400mg/day, W6-W12: 200mg/day for jaundice | 1 | 1 |  |
|  |  | W1-W4: 600mg/day, W5-W12: 200mg/day for anemia | 1 | 0 | Attained ETR, but died at post-treatment week 7 due to suspected acute myocardial infarction |
|  |  | W1-W2: 1000mg/day, W3-W4: 600mg/day, W5-W12: 400mg/day for anemia | 1 | 1 |  |
| C | No | 800mg/day | 1 | 1 |  |
